# Third generation cephalosporin resistant *Enterobacteriaceae* and multidrug resistant gram-negative bacteria causing bacteremia in febrile neutropenia adult cancer patients in Lebanon, broad spectrum antibiotics use as a major risk factor, and correlation with poor prognosis

**DOI:** 10.3389/fcimb.2015.00011

**Published:** 2015-02-12

**Authors:** Rima Moghnieh, Nour Estaitieh, Anas Mugharbil, Tamima Jisr, Dania I. Abdallah, Fouad Ziade, Loubna Sinno, Ahmad Ibrahim

**Affiliations:** ^1^Department of Internal Medicine, Makassed General HospitalBeirut, Lebanon; ^2^Division of Hematology-Oncology, Department of Internal Medicine, Makassed General HospitalBeirut, Lebanon; ^3^Department of Laboratory Medicine, Makassed General HospitalBeirut, Lebanon; ^4^Pharmacy Department, Makassed General HospitalBeirut, Lebanon; ^5^Faculty of Public Health, Lebanese UniversityBeirut, Lebanon; ^6^Research Coordinator, Makassed General HospitalBeirut, Lebanon

**Keywords:** febrile neutropenia, bacteremia, 3GCR gram-negative bacteria, MDR gram-negative bacteria, Lebanon

## Abstract

**Introduction:** Bacteremia remains a major cause of life-threatening complications in patients receiving anticancer chemotherapy. The spectrum and susceptibility profiles of causative microorganisms differ with time and place. Data from Lebanon are scarce. We aim at evaluating the epidemiology of bacteremia in cancer patients in a university hospital in Lebanon, emphasizing antibiotic resistance and risk factors of multi-drug resistant organism (MDRO)-associated bacteremia.

**Materials and Methods:** This is a retrospective study of 75 episodes of bacteremia occurring in febrile neutropenic patients admitted to the hematology-oncology unit at Makassed General Hospital, Lebanon, from October 2009-January 2012. It corresponds to epidemiological data on bacteremia episodes in febrile neutropenic cancer patients including antimicrobial resistance and identification of risk factors associated with third generation cephalosporin resistance (3GCR) and MDRO-associated bacteremia.

**Results:** Out of 75 bacteremias, 42.7% were gram-positive (GP), and 57.3% were gram-negative (GN). GP bacteremias were mostly due to methicillin-resistant coagulase negative staphylococci (28% of total bacteremias and 66% of GP bacteremias). Among the GN bacteremias, *Escherichia coli* (22.7% of total, 39.5% of GN organisms) and *Klebsiella pneumoniae*(13.3% of total, 23.3% of GN organisms) were the most important causative agents. GN bacteremia due to 3GC sensitive (3GCS) bacteria represented 28% of total bacteremias, while 29% were due to 3GCR bacteria and 9% were due to carbapenem-resistant organisms. There was a significant correlation between bacteremia with MDRO and subsequent intubation, sepsis and mortality. Among potential risk factors, only broad spectrum antibiotic intake >4 days before bacteremia was found to be statistically significant for acquisition of 3GCR bacteria. Using carbapenems or piperacillin/tazobactam>4 days before bacteremia was significantly associated with the emergence of MDRO (*p* < 0.05).

**Conclusion:** Our findings have major implications for the management of febrile neutropenia, especially in breakthrough bacteremia and fever when patients are already on broadspectrum antibiotics. Emergence of resistance to 3GCs and, to a lesser extent, to carbapenems in GN isolates has to be considered seriously in our local guidelines for empiric treatment of febrile neutropenia, especially given that their occurrence was proven to be associated with poorer outcomes.

## Introduction

The progress of anticancer therapy with aggressive supportive care for patients with malignancies and patients undergoing hematopoietic stem cell transplantation (HSCT) have recently improved patient prognosis (Trecarichi and Tumbarello, 2014). However, these advances, resulting in a prolonged and profound level of immunosuppression, neutropenia in particular, along with the extensive use of implantable medical devices, have also increased the risk of severe infections (Trecarichi and Tumbarello, 2014). Different types of infections may occur in cancer patients, but bloodstream infections (BSIs) are the most common severe infectious complications; the reported prevalence of BSIs ranges from 11 to 38%, and the crude mortality rate reaches up to 40% (Wisplinghoff et al., 2003a,b; Tumbarello et al., 2012; Montassier et al., 2013).

The type of microorganisms isolated on blood culture from febrile neutropenic patients varies with time and place (Jones, 1999; Dettenkofer et al., 2003; Wisplinghoff et al., 2003b; Irfan et al., 2008; Freifeld et al., 2011). Data from the Middle East and North Africa (MENA) region is scarce.

At the beginning of the use of cytotoxic chemotherapy in the 1960s and 1970s in cancer patients, gram-negative bacteria (GNB) were the most common organisms causing bacteremia in febrile neutropenic patients (Jones, 1999; Irfan et al., 2008); however, at the turn of the century, the most common bacterial pathogens isolated from blood cultures were coagulase-negative staphylococci (Dettenkofer et al., 2003; Wisplinghoff et al., 2003b; Freifeld et al., 2011).

However, in recent years several studies have demonstrated a clear trend in the epidemiology of BSIs, showing a shift of prevalence from gram-positive to gram-negative bacteria (Wisplinghoff et al., 2003b; Pagano et al., 2012; Montassier et al., 2013). There is an emergence of drug-resistant GNB such as multidrug resistant (MDR) *Pseudomonas aeruginosa*, *Acinetobacter baumannii*, *Stenotrophomonas maltophilia*, extended-spectrum beta-lactamase (ESBL)-producing GNB, and carbapenemase-producing GNB (Zinner, 1999; Wisplinghoff et al., 2003b; Ramphal, 2004; Freifeld et al., 2011; Pagano et al., 2012; Wu et al., 2012; Montassier et al., 2013). The issue of antimicrobial resistance has become a significant problem worldwide, where treatment of infections due to MDR bacteria represents a clinical challenge because the therapeutic options are often very limited. Risk factors of bacteremia due to MDR GNB in febrile neutropenic cancer patients vary depending on the type of organism, duration of hospitalization, and antibiotic therapy (Gudiol and Carratala, 2014).

So, the fact that the epidemiology of pathogens is dynamic makes contemporary local data extremely important, and identification of pathogens locally recovered from blood cultures of febrile neutropenic patients and the patterns of their antibiotic susceptibilities are essential in making therapeutic decisions (Sigurdardottir et al., 2005).

The purpose of this study is to evaluate the epidemiology of bacteremia occurring during neutropenia in adult cancer chemotherapy patients in a university hospital in Lebanon, with a special emphasis on the prevalence, susceptibility profile and risk factors associated with bacteremia caused by third generation cephalosporin-resistant (3GCR) and carbapenem-resistant bacteria.

## Materials and methods

### Setting, patients and study design

This is retrospective study performed at Makassed General Hospital, a 200-bed university hospital in Beirut, Lebanon. The hospital's Institutional Review Board approved the study, and an informed consent was waived since it was observation. Medical records of patients admitted between October 2009 and January 2012 were reviewed. Seventy-five episodes of bacteremia occurring in 70 hospitalized neutropenic adult patients were recorded.

Adult cancer patients with fever and neutropenia, including those undergoing Hematopoietic Stem Cell Transplantation (HSCT), with positive blood cultures were selected. All positive results of blood cultures with the corresponding antibiogram were checked and recorded from the Microbiology Laboratory log books and computerized laboratory records. Information regarding these episodes were collected and recorded in a specific database from patients' medical records.

Patient characteristics were identified, including age, gender, type of cancer, risk level, recurrence of admissions, duration of neutropenia prior to bacteremia, hospital stay prior to bacteremia, presence of a focus of infection, presence of a central line or an implantable venous access, outcome, sepsis, intubation, HSCT, antibiotic and antifungal intake in the hospital setting. Univariate analysis of these characteristics was used to identify risk factors for3GCR and multi-drug resistant (MDR) organism-associated bacteremia.

### Definitions and inclusion criteria

Patients were included if they met all three of the following inclusion criteria:

1. Fever, defined as a single oral temperature of 38.3°C or an oral temperature of 38°C lasting 1 h or more.
2. Neutropenia, defined as a neutrophil count of <500 cells/mm^3^, or a count of <1000 cells/mm^3^ with a documented decrease to <500 cells/mm^3^ within the following 48–72 h.
3. Receipt of chemotherapy prior to the episode of febrileneutropenia.

Patients who had fever and neutropenia as a result of their underlying disease without having received chemotherapy were excluded.

In Lebanon, anaerobic cultures are performed only in reference laboratories. Hospital-based laboratories perform only aerobic cultures. According to hospital policy, one set of blood cultures consists of two bottles of aerobic cultures taken from two different draws at the same time, with no bottles for anaerobic culture. Bacteremia is defined as isolation of the same bacterial or pathogen from at least one set of blood cultures (2 bottles taken at the same time). Bacteremia is considered polymicrobial if at least two organisms from the same blood culture on two occasions are isolated or more than one organism each in at least two separate blood cultureswithin 48 h (Reuben et al., 1989). Bacteremia occurring more than 14 days after a previous episode and separated by repeatedly negative blood cultures was considered a separate episode. Each separate hospital admission for febrile neutropenia was defined as one episode. Subsequent hospital admissions for febrile neutropenia in the same patient were included as separate cases.

Bacteremia caused by a potential skin contaminant (such as coagulase-negative staphylococci, *Bacillus*, or *Corynebacterium* species) was considered significant only if it met the following criteria:

1. Growth of the same bacterial strainin two blood cultures taken from two different sites at the same time.
2. Growth of the same bacterial strain in one blood culture and in one other sterile site (urine, cerebrospinal fluid, ascetic fluid, pleural fluid, joint fluid).
3. Growth of the same bacterial strain in one peripheral blood culture and one blood culture taken from an intravenous catheter where both cultures were taken at the same time.

Interpretive criteria (breakpoints) for susceptible, intermediate, and resistant bacterial isolates were those included in the Clinical and Laboratory Standards Institute guidelines (Clinical and Laboratory Standards Institute, 2010).

The 3GCR *Enterobacteriaceae* phenotypes included all isolates not susceptible to one or more of five agents including aztreonam, cefotaxime, ceftizoxime, ceftazidime, and ceftriaxone (Clinical and Laboratory Standards Institute, 2010). These isolates were, however, susceptible to imipenem.

Many different definitions for multidrug resistance are used in the medical literature to characterize different patterns of resistance in healthcare-associated, antimicrobial-resistant bacteria (Magiorakos et al., 2012).However, generally speaking, MDR-Gram negative bacteria are resistant to key antimicrobial agents (Siegel et al., 2007; Hidron et al., 2008). Third and fourth generation cephalosporins, along with fluoroquinolones, aminoglycosides, and carbapenems, constitute the major therapeutic options in treatment guidelines of febrile neutropenia in adult cancer patients (Freifeld et al., 2011; Averbuch et al., 2013). In this study, gram-negative bacteria were considered MDR when resistant to third and fourth generation cephalosporins, fluoroquinolones, aminoglycosides and carbapenems, including *S. maltophilia*, carbapenem-resistant *P. aeruginosa* and *Acinetobacter baumannii*, or carbapenem-resistant 3GCR *Enterobacteriaceae.*

### Statistical analysis

Data were reported as the mean standard deviation (SD) or number of patients (percentage). *T*-tests (two-tailed), Fisher's exact tests and Chi-square tests were used to assess any significant differencesamong the groups. *P* < 0.05 were considered statistically significant.

## Results

During the study period, 75 episodes of bacteremia occurring in 70 hospitalized neutropenic adult patients with hematological malignancies, including those undergoing HSCT, were recorded. Epidemiological and clinical characteristics of the patients, antibiotic treatment, and patient outcomes are shown in Table 1.

**Table 1:** **Epidemiological and clinical characteristics of all episodes of bacteremia**.

**Characteristic**	**Total [***n*** = **75**(%)]**
**AGE (YEARS)**	
0–18	5 (6.67)
18–65	62 (82.67)
>65	8 (10.67)
**GENDER**	
Male	36 (48)
Female	39 (52)
**TUMOR TYPE**	
Leukemia	56 (74.67)
Lymphoma	15 (20)
Solid	4 (5.33)
MASCC score	
>21	5 (6.67)
<21	70 (93.33)
**NEUTROPENIA DURATION PRIOR TO BACTEREMIA**	
<7 days	26 (34.67)
>7 days	26 (34.67)
Unknown	23 (30.67)
**HOSPITAL STAY PRIOR TO BACTEREMIA**	
<2 days	21 (28)
Between 2 and 7 days	4 (5.33)
>7 days	48 (64)
Unknown	2 (2.67)
**RECURRENT ADMISSION**	
Yes	67 (89.33)
No	8 (10.67)
**FOCUS OF INFECTION**	
No/Unknown	17 (22.67)
Pneumonia	3 (4)
Gastroenteritis	13 (17.33)
Urinary tract infection	7 (9.33)
Skin and soft tissue infection	3 (4)
Central line associated	30 (40)
Portal catheter associated	2 (2.67)
**PLACEMENT OF CENTRAL VENOUS CATHETER**	
Yes	52 (69.33)
<10 days	18 (24)
>10 days	34 (45.33)
No	23 (30.67)
**OUTCOME**	
Death	7 (9.33)
Recovery	68 (90.67)
Sepsis	8 (10.67)
Intubation	7 (9.33)
Hematopoietic stem cell transplantation	41 (54.67)

N.B. MASCC, Multinational Association of Supportive Care in Cancer.

Of the 75 bacteremias, 42.7% were due to gram-positive organisms, and the remaining 57.3% were gram-negative. Gram-positive bacteremias were mostly due to methicillin-resistant coagulase negative staphylococci, which represented 66% of gram-positive bacteremias and 28% of total bacteremias. No methicillin-resistant *Staphylococcus aureus*-related bacteremia was detected and only one episode of methicillin-sensitive *Staphylococcus aureus*-related bacteremia was observed. (Refer to Tables 2, 3).

**Table 2:** **Causative organisms of all episodes of bacteremia**.

**Causative organisms**	**Number of episodes [***n*** = **75**(%)]**
gram-positive Bacteria	32 (42.67)
*Staphylococcus aureus*	1 (1.33)
*Coagulase-negative staphylococci* (CNS)	28 (37.33)
Methicillin resistant CNS	21 (28)
*Streptococcus pneumoniae*	1 (1.33)
*Enterococcus faecium*	1 (1.33)
*Aerococcus viridans*	1 (1.33)
gram-negative Bacteria (GNB)	43 (57.33)
*Escherichia coli*	17 (22.67)
3GCR -*Escherichia coli*	8 (10.67)
*Pseudomonas aeruginosa*	3 (4)
*Pseudomonas putida*	1 (1.33)
*Pseudomonas stutzeri*	1 (1.33)
*Klebsiella pneumoniae*	10 (13.33)
3GCR- *Klebsiella pneumoniae*	5 (6.67)
*Klebsiella oxytoca*	1 (1.33)
*Proteus mirabilis*	3 (4)
*Enterobacter cloacae*	2 (2.67)
*Acinetobacter baumannii*	2 (2.67)
*Stenotrophomonas maltophilia*	2 (2.67)
*Salmonella species*	1 (1.33)
3GC sensitive GNB	21 (28)
3GCR GNB	22 (29.33)
3GCR carbapenem-sensitive GNB	15 (20)
3GCR carbapenem-resistant GNB	7 (9.33)

N.B. 3GCR, third generation cephalosporin-resistant.

**Table 3:** **Antibiotic susceptibility profile of isolated gram-positive bacteria**.

**Antibiotic**	**No of susceptible gram-positive isolates (%)**					
	**CNS [***n*** = **28**(%)]**	***S. aureus* [***n*** = **1**(%)]**	***S. pneumoniae* [***n*** = **1**(%)]**	***E. faecium* [***n*** = **1**(%)]**	***A. viridans* [***n*** = **1**(%)]**	**Total [***n*** = **32**(%)]**
Oxacillin	7 (25)	1 (100)	1 (100)	0 (0)	0 (0)	9 (28.1)
Ampicillin	–	1 (100)	1 (100)	0 (0)	0 (0)	10 (31.3)
Rifampin	24 (85.7)	1 (100)	1 (100)	0 (0)	0 (0)	26 (81.3)
Clindamycin	16 (57.1)	1 (100)	1 (100)	0 (0)	1 (100)	19 (59.4)
Teicoplanin	28 (100)	1 (100)	1 (100)	1 (100)	1 (100)	32 (100)
Vancomycin	28 (100)	1 (100)	1 (100)	1 (100)	1 (100)	32 (100)

N.B. A.viridans, Aerococcus viridans; CNS, Coagulase Negative Staphylococci; E. faecium, Enterococcus faecium; S. aureus, Staphylococcus aureus; S. pneumoniae, Streptococcus pneumonia.

Among the gram-negative bacteremias, *Escherichia coli* (22.7% of total, 39.5% of gram-negative) and *Klebsiella pneumonia* (13.3% of total, 23.3% of gram-negative) were the most important causative agents. Out of the 17 bacteremias caused by *E. coli*, eight were due to 3GCR resistant strains (10.7% of total bacteremias and 47% of *E. coli* strains). From those caused by *K. pneumoniae* (10 bacteremias), five cases were due to 3GCR strains (6.7% of total bacteremia and 50% of *K. pneumoniae*). In general, 28% of the total bacteremias were due to 3GC sensitive gram-negative bacteria and 29.3% of the total bacteremias were caused by 3GC resistant gram-negative bacteria. Concerning carbapenem susceptibility in the 3GC resistant category, seven cases were carbapenem-resistant, representing 9.3% of total bacteremias. (Refer to Tables 2, 4).

**Table 4:** **Antibiotic susceptibility profile of isolated gram-negative bacteria**.

**No. of susceptible gram-negative isolates (%)**									
**Antibiotic**	***E. coli* [***n*** = **17**(%)]**	***Klebsiella* spp. [***n*** = **11**(%)]**	***Pseudomonas* spp. [***n*** = **5**(%)]**	***P.mirabilis* [***n*** = **3**(%)]**	***Enterobacter* spp. [***n*** = **2**(%)]**	***S. maltophilia* [***n*** = **2**(%)]**	***A. baumannii* [***n*** = **2**(%)]**	***Salmonella* spp. [***n*** = **1**(%)]**	**Total [***n*** = **43**(%)]**
Amox/Clav	5 (29.4)	5 (45.5)	0 (0)	3 (100)	0 (0)	0 (0)	0 (0)	1 (100)	11 (25.6)
Amikacin	15 (88.2)	10 (90.9)	5 (100)	3 (100)	2 (100)	0 (0)	1 (50)	1 (100)	37 (86)
Cefepime	5 (29.4)	6 (54.5)	3 (60)	3 (100)	1 (50)	0 (0)	0 (0)	1 (100)	19 (44.2)
Ceftazidime	8 (57.1)	5 (45.5)	4 (80)	3 (100)	0 (0)	1 (50)	1 (50)	1 (100)	23 (53.5)
Ceftriaxone	8 (57.1)	6 (54.5)	2 (40)	3 (100)	0 (0)	0 (0)	0 (0)	1 (100)	19 (44.2)
Imipenem	14 (82.4)	11 (100)	4 (80)	3 (100)	2 (100)	0 (0)	1 (50)	1 (100)	36 (83.7)
Pip/Tazo	13 (76.5)	7 (63.6)	5 (100)	3 (100)	0 (0)	0 (0)	1 (50)	1 (100)	30 (69.8)
Tigecycline	17 (100)	10 (90.9)	1 (20)	3 (100)	2 (100)	2 (100)	2 (100)	1 (100)	40 (93)
Colistin	17 (100)	11 (100)	4 (80)	3 (100)	2 (100)	0 (0)	2 (100)	1 (100)	40 (93)
Quinolones	7 (41.2)	7 (63.6)	4 (80)	1 (33.3)	0 (0)	2 (100)	1 (50)	0 (0)	22 (51.2)

N.B. A.baumannii, Acinetobacter baumannii; Amox/Clav, Amoxicillin/Clavulanate; E. coli, Escherichia coli; Pip/Tazo, Piperacillin/Tazobactam; P.mirabilis, Proteus mirabilis; S. maltophilia, Stenotrophomonas maltophilia; spp, species.

Results of the univariate analysis of factors potentially associated with 3GCR bacteremia, including baseline and demographic characteristics, major disease and risk, hospitalization prior to bacteremia, recurrent admissions, neutropenia prior to bacteremia, presence of CVC, and focus of infection, did not show any statistical significance, suggesting that the former factors were not risk factors. However, history and duration of antibiotic intake before the episode of bacteremia was majorly implicated in the occurrence of 3GCR bacteremia. The type of broad spectrum antibiotic use did not affect the results; butduration of intake did affect results. The use of carbapenems, piperacillin/tazobactam, or 3rd or 4th GC ± aminoglycosides for more than 4 days prior to the bacteremic episode was significantly associated with 3GCR bacteremia compared with all other types of bacteremias. (*P* < 0.01) A worse outcome, defined by need for intubation and the occurrence of sepsis in bacteremic patients, was also statistically significant in the 3GCR group compared with the other groups. (*P* < 0.03) (Refer to Tables 5, 6).

**Table 5:** **Baseline and demographic characteristics, clinical features, and outcome of patients with 3GCR-bacteremia compared with non-3GCR-bacteremia and MDR-bacteremia compared with non-MDR-bacteremia**.

**Characteristic**	**3GCR-Bacteremia [***n*** = **22**(%)]**	**Non-3GCR-Bacteremia [***n*** = **53**(%)]**	***P*-value**	**MDR Bacteremia [***n*** = **7**(%)]**	**Non-MDR Bacteremia [***n*** = **68**(%)]**	***P*-value**
**AGE (YEARS)**						
0–18	1 (4.5)	4 (7.5)		0 (0)	5 (7.4)	
18–65	19 (86.4)	43 (81.1)		6 (85.7)	56 (82.4)	
>65	2 (9.1)	6 (11.3)	0.846	1 (14.3)	7 (10.3)	0.734
**GENDER**						
Male	9 (40.9)	27 (50.9)		3 (42.9)	33 (48.5)	
Female	13 (59.1)	26 (49.1)	0.428	4 (57.1)	35 (51.5)	0.775
**TUMOR TYPE**						
Leukemia	17 (77.3)	39 (73.6)		6 (85.7)	50 (73.5)	
Lymphoma	4 (18.2)	11 (20.8)		0 (0)	15 (22.1)	
Solid	1 (4.5)	3 (5.7)	0.944	1 (14.3)	3 (4.4)	0.243
**MASCC SCORE**						
>21	0 (0)	5 (9.4)		0 (0)	5 (7.4)	
<21	22 (100)	48 (90.6)	0.136	7 (100)	63 (92.6)	0.458
**NEUTROPENIA DURATION PRIOR TO BACTEREMIA**						
<7 days	8 (36.4)	15 (28.3)		3 (42.9)	20 (29.4)	
>7 days	7 (31.8)	19 (35.8)		1 (14.3)	25 (36.8)	
Unknown	7 (31.8)	19 (35.8)	0.789	3 (42.9)	23 (33.8)	0.485
**HOSPITAL STAY PRIOR TO BACTEREMIA**						
<2 days	7 (31.8)	14 (26.4)		2 (28.6)	19 (27.9)	
Between 2 and 7 days	2 (9.1)	2 (3.8)		1 (14.3)	3 (4.4)	
>7 days	13 (59.1)	35 (66)		4 (57.1)	44 (64.7)	
Unknown	0 (0)	2 (3.8)	0.586	0 (0)	2 (2.9)	0.700
**RECURRENT ADMISSION**						
Yes	2 (9.1)	6 (11.3)		0 (0)	8 (11.8)	
No	20 (90.9)	47 (88.7)	0.776	7 (100)	60 (88.2)	0.337
**FOCUS OF INFECTION**						
No/Unknown	3 (13.6)	14 (26.4)		1 (14.3)	16 (23.5)	
Pneumonia	1 (4.5)	2 (3.8)		1 (14.3)	2 (2.9)	
Gastroenteritis	0 (0)	3 (5.7)		0 (0)	3 (4.4)	
UTI	6 (27.3)	7 (13.2)		2 (28.6)	11 (16.2)	
SStI	8 (36.4)	22 (41.5)		2 (28.6)	28 (41.2)	
Central line associated	1 (4.5)	1 (1.9)		0 (0)	2 (2.9)	
Portal catheter associated	3 (13.6)	4 (7.5)	0.507	1 (14.3)	6 (8.8)	0.702
**PLACEMENT OF CVC**						
Yes	7 (31.8)	16 (30.2)		2 (28.6)	21 (30.9)	
<10 days	6 (27.3)	12 (22.6)		3 (42.9)	15 (22.1)	
>10 days	9 (40.9)	25 (47.2)		2 (28.6)	32 (47.1)	
No	7 (31.8)	16 (30.2)	0.866	2 (28.6)	21 (30.9)	0.442
**OUTCOME**						
Death	5 (22.7)	2 (3.8)		4 (57.1)	3 (4.4)	
Recovery	17 (77.3)	51 (96.2)	0.010	3 (42.9)	65 (95.6)	<0.0001
Sepsis	5 (22.7)	3 (5.7)	0.029	4 (57.1)	4 (5.9)	<0.0001
Intubation	5 (22.7)	2 (3.8)	0.010	4 (57.1)	3 (4.4)	<0.0001
HSCT	13 (59.1)	29 (54.7)	0.728	4 (57.1)	38 (55.9)	0.949

N.B. MASCC, Multinational Association of Supportive Care in Cancer, UTI, Urinary tract infection; SStI, Skin and soft tissue Infection; CVC, Central Venous Catheter; HSCT, Hematopoietic stem cell transplantation.

**Table 6:** **Antimicrobial history and duration prior to bacteremia in all cases, in patients with 3GCR- bacteremia compared with non-3GCR-bacteremia and in MDR-bacteremiacompared with non-MDR-bacteremia**.

**Antimicrobial duration before bacteremia**	**Total ***n*** = **75** (%)**	**3GCR-Bacteremia [***n*** = **22**(%)]**	**Non-3GCR-Bacteremia [***n*** = **53**(%)]**	***P*-value**	**MDR-Bacteremia [***n*** = **7**(%)]**	**Non-MDR-Bacteremia [***n*** = **68**(%)]**	***P*-value**
Carbapenem (Group1) <4 days	8 (10.67)	1 (4.5)	7 (13.2)	0.269	1 (14.3)	7 (10.3)	0.745
Carbapenem>4 days	14 (18.67)	8 (36.4)	6 (11.3)	0.011	4 (57.1)	10 (14.7)	0.006
No Carbapenem	55 (73.33)	1 (4.5)	4 (7.5)	0.635	1 (14.3)	4 (5.9)	0.396
PIP/TAZ (Group2) <4 days	5 (6.67)	5 (22.7)	2 (3.8)	0.010	2 (28.6)	5 (7.4)	0.066
PIP/TAZ >4 days	7 (9.33)	3 (13.6)	12 (22.6)	0.375	3 (42.9)	12 (17.6)	0.112
No PIP/TAZ	65 (86.67)	6 (27.3)	5 (9.4)	0.047	2 (28.6)	9 (13.2)	0.275
3rd or 4th GC ± Amikacin (Group3) <4 days	15 (20)	3 (13.6)	12 (22.6)	0.375	3 (42.9)	12 (17.6)	0.112
3rd or 4th GC ± Amikacin>4 days	11 (14.67)	8 (36.4)	6 (11.3)	0.011	3 (42.9)	11 (16.2)	0.085
No 3rd or 4th GC ± Amikacin	51 (68)	2 (9.1)	11 (20.8)	0.224	2 (28.6)	11 (16.2)	0.409
Group 1 or 2 or 3 <4 days	15 (20)	7 (31.8)	5 (9.4)	0.016	3 (42.9)	9 (13.2)	0.042
Group 1 or 2 or 3 >4 days	14 (18.67)	2 (9.1)	5 (9.4)	0.963	2 (28.6)	5 (7.4)	0.066
No group 2 or 3 or 4	49 (65.33)	4 (18.2)	5 (9.4)	0.288	2 (28.6)	7 (10.3)	0.156
Carbapenem or PIP/TAZ <4 days	13 (17.33)	1 (4.5)	7 (13.2)	0.269	1 (14.3)	7 (10.3)	0.745
Carbapenem or PIP/TAZ >4 days	12 (16)	8 (36.4)	6 (11.3)	0.011	4 (57.1)	10 (14.7)	0.006
No Carbapenem or PIP/TAZ	52 (69.33)	1 (4.5)	4 (7.5)	0.635	1 (14.3)	4 (5.9)	0.396
Antifungal other than Fluconazole <4 days	7 (9.33)	5 (22.7)	2 (3.8)	0.010	2 (28.6)	5 (7.4)	0.066
Antifungal other than Fluconazole >4 days	9 (12)	3 (13.6)	12 (22.6)	0.375	3 (42.9)	12 (17.6)	0.112
No Antifungal other than Fluconazole	62 (82.67)	6 (27.3)	5 (9.4)	0.047	2 (28.6)	9 (13.2)	0.275

N.B. PIP/TAZ, Piperacillin/Tazobactam; 3rd or 4th GC, Third or Fourth Generation Cephalosporin.

Similarly, infection with an MDR strain was associated with significantly higher rates of subsequent intubation, sepsis, and mortality. (*P* < 0.03) The history and duration of antibiotic intake before the episode of bacteremia was significantly associated with the occurrence of MDR bacteremia as well. The use of cephalosporins ± aminoglycosides was not significantly associated with MDR bacteremia, while the use of carbapenems or piperacillin/tazobactam for more than 4 days prior to MDR-bacteremia was significantly associated with its occurrence. (*P* < 0.04) (Refer to Tables 5, 6).

## Discussion

Data from Lebanon in neutropenic patients is scarce, and the available literature reveals the dynamic nature of the etiology of bacteremias with time. Previous studies from Lebanon have shown gram-negative organisms to be the predominant agents in febrile neutropenic patients (Hamzeh et al., 2000; Kanafani et al., 2007). In addition to the subdivision between gram-positive and gram-negative organisms, we investigated antibiotic susceptibility patterns and the risk factors associated with bacteremia caused by antibiotic-resistant gram-negative organisms.

In this study, the distribution of the 75 cases was almost equal between gram-positive and gram-negative organisms (42.7 vs. 57.3%). Yet, a study by Kanafani et al. (2007) in 2007 from Lebanon showed gram-negative predominance in febrile neutropenia bacteremic episodes. Gram-negative organisms were responsible for 78.8% of bloodstream infections compared with 33.3% gram-positive organisms (Kanafani et al., 2007).

In fact, the shift from a preponderance of gram-negative bacteria causing bloodstream infections in febrile neutropenic patients to gram-positive bacteria has been observed worldwide. The widespread use of indwelling catheters, early-generation fluoroquinolone prophylaxis, and broad spectrum empirical anti-gram-negative antibacterial therapy led to an increase in the incidence of gram-positive pathogens in the 1980s and 1990s (Zinner, 1999; Ramphal, 2004; Freifeld et al., 2011). Thereafter, the most common bacterial etiologic agent isolated from blood cultures in most centers was reported to be coagulase-negative staphylococci (Dettenkofer et al., 2003; Wisplinghoff et al., 2003b; Freifeld et al., 2011). In our study, coagulase-negative staphylococci were the most common among gram-positive organisms (37.3% of total cases, 87.5% of gram-positive cases), where methicillin-resistant coagulase-negative staphylococciisolatesrepresented 28% of the total cases and 65.6% of gram-positive cases.

Our results showed that *E. coli* and *K. pneumoniae* were the most prevalent gram-negative organisms, representing 22.7 and 13.3%, respectively, of total cases. A systematic literature review conducted by Trecarichi and Tumbarello (2014) from January 2007 to August 2013 examined the recent trends in epidemiology and antimicrobial resistance in gram-negative bacteria recovered from cancer patients, with a particular emphasis on the impact of antimicrobial resistance on the clinical outcome of severe infections caused by such microorganisms. The gram-negative bacterial species most frequently isolated were *E. coli*, whose frequency ranged from 10.1 to 53.6% (mean 32.1%), and *K. pneumoniae*, which was isolated with a frequency ranging from 4.1 to 44.6% (mean 19.5%) (Trecarichi and Tumbarello, 2014).

In our study, 3GCR strains of *E. coli* and *K. pneumoniae* caused 10.7 and 6.7% of total bacteremias, respectively. 3GCR-*Enterobacteriaceae* colonization or infection of patients with febrile neutropenia has been reported with increased frequency during the last decade. Bloodstream infections due to 3GCR *E. coli* isolates have been reported with a frequency ranging from 12 to 75% (mean 35%) in cancer patients. 3GCR *K. pneumoniae* isolates causing BSIs in neutropenic patients have been reported with a frequency ranging from 3 to 66.6% (mean 37.8%) (Trecarichi and Tumbarello, 2014).

We found that 9.3% of episodes of bacteremia in cancer patients were caused by MDR gram-negative bacteria. This finding is in line with recent studies, which report an increase in antibiotic resistance among gram-negative bacteria in immunocompromised hosts. One study performed in Italy by Gudiol et al. (2011) reported an incidence of 13.7% of MDR gram-negative associated bacteremia, andin another study performed in Pakistan by Irfan et al. (2008), the emergence of carbapenem resistance was reported in *Pseudomonas* species (20.7% of the isolates) and in *Acinetobacter* species (65.4% of the isolates). In another study by Trecarichi et al. (2011) from Italy, among 38 patients diagnosed with *P. aeruginosa* bacteremia, 27 were MDR species (71.1%). The percentages of *in vitro* resistance to major antimicrobial classes were the following: carbapenems (imipenem and meropenem) 60%, antipseudomonal cephalosporins (ceftazidime and cefepime) 42%, and piperacillin 24% (Trecarichi et al., 2011).

The emergence of 3GCR *Enterobacteriaceae* and MDR gram-negative organisms causing bacteremia in cancer patients is very critical in terms of empiric therapy for febrile neutropenia. Third and fourth generation cephalosporins remain the first line option for primary therapy in febrile neutropenia (Freifeld et al., 2011; Averbuch et al., 2013). In this group of patients, appropriate initial empirical antibiotic therapy is essential, (Gudiol and Carratala, 2014) and empiric antibiotic therapy in febrile neutropenic patients has reduced mortality rates from approximately 21% (Viscoli et al., 2005) to 2–10%, (Vidal et al., 2004; Toussaint et al., 2006) depending upon the underlying diagnosis, degree of cancer control, duration of severe neutropenia and type of infection. Inappropriate empiric therapy is defined, in context, as not including at least one antibiotic active *in vitro* against the infecting microorganism(s) (Freifeld et al., 2011; Averbuch et al., 2013), and the emergence of 3GCR and MDR organisms in febrile neutropenia renders 3rd or 4th GCs inappropriate choices in certain situations.

Our results showed that the use of broad spectrum antibiotics, including carbapenems, piperacillin/tazobactam, and 3rd or 4th GC ± aminoglycosides, for more than 4 days prior to bacteremia was significantly associated with 3GCR bacteremia. (*P* < 0.01) However, in the case of carbapenem-resistant 3GCR bacteremia (MDR), the use of carbapenems or piperacillin/tazobactam, but not cephalosporins, for more than 4 days prior to MDR-bacteremia was significantly associated with its occurrence. (*P* < 0.04) (Refer to Table 6) Previous antibiotic therapy has been recognized as a major risk factor for the development of bacterial resistance. In prospective study involving13 Brazilian HSCT centers (Oliveira et al., 2007), 22% of 91 episodes of bacteremia were MDR-gram-negative in origin. Previous exposure to third generation cephalosporins either as prophylaxis or empirical therapy and belonging to one of the HSCT centers were associated with an increased risk for ESBL-producing *Enterobacteriaceae* (Oliveira et al., 2007). Another retrospective case-control study involving HSCT recipients (Garnica et al., 2009) showed by univariate analysis that previous use of a third or fourth-generation cephalosporin (*P* = 0.005 and 0.02, respectively) and duration of antibiotic use (*P* < 0.001) were among the factors associated with bacteremia due to MDR-gram-negative isolates including *K. pneumonia* and *P. aeruginosa*. In another study performed in the United States by Rangaraj et al. (2010), the use of multiple broad spectrum antibiotics compared with no antimicrobial agents was significantly associated with isolation of MDR *P. aeruginosa* (8.2 vs. 0.7%, *p* < 0.005). This finding is consistent with a more recent study in 2013 by Satlin et al. (2013), where exposure to any broadspectrum antibacterial agent may be sufficient to increase the risk of carbapenem-resistant *Enterobacteriaceae* acquisition and cause bloodstream infections in patients with hematologic malignancies.

Our results showed that patient outcome was influenced significantly by antimicrobial resistance, and the risk ofsubsequent intubation, sepsis and mortality were high in the 3GC-resistant bacteremia group and in the MDR-bacteremia group compared with patients having other bacteremias(*P* < 0.03). (Refer to Table 5) Other studies indicated that a dramatic increase in the detection rate of MDR gram-negative bacteremia compared with previous periods was associated with increased morbidity, mortality, and cost, especially in patients with hematological diseases (Lodise et al., 2007). Moreover, mortality was independently associated with inadequate initial antimicrobial treatment in the case of antibiotic-resistant bacteremia (Giske et al., 2008). Thus, local monitoring of bacterial isolates is recommended to adapt initial empiric antibiotic therapy based on the local prevalence of MDR strains (Caselli et al., 2010).

Our study has at least two major limitations. The first is that the samples were collected from a single medical center; therefore, results could not be generalized to other medical centers in Lebanon because the microbial ecology differs from one center to another. The second limitation is that the small sample size did not allow us to perform a multivariate analysis and limited our statistical analysis to a univariate model.

## Conclusion

In conclusion, our data showed equal occurrence of gram-negative and gram-positive organisms causing bacteremia in febrile neutropenic cancer patients in our center. We found that bacteremia caused by gram-negative antimicrobial resistant strains is common among cancer patients, especially in those exposed to antibiotic pressure. Emergence of resistance to third and fourth generation cephalosporins and, to a lesser extent, to carbapenems, in gram-negative isolates has to be considered seriously in our local guidelines for empiric treatment of febrile neutropenia, especially given that their occurrence was associated with poorer clinical outcomes.

The empiric use of broad spectrum antibiotics in febrile neutropenia is very critical. On the one hand, it is crucial to decrease mortality during the febrile episode; on the other hand, it is a risk factor for emergence bacteremia with resistant organisms. In our therapeutic guidelines for the management of febrile neutropenia, we should include coverage for MDR bacteria in patients who have persistent or relapsing fever after 4 days of initial empiric therapy.

## Author contributions

All authors have contributed equally to the analysis and interpretation of the study data as well as to the drafting of the article, but Rima Moghnieh made the primary contribution to the conception and design of the study and revising the draft critically for important intellectual contentand Nour Estaitieh to the acquisition and collection of data. All authors gave the final approval of the article to be sent for publication and agreed to be accountable for all aspects of the paper in ensuring that questions related to the accuracy or integrity of any part of the work are appropriately investigated and resolved.

### Conflict of interest statement

The authors declare that the research was conducted in the absence of any commercial or financial relationships that could be construed as a potential conflict of interest.
